# Letter to the editor: A norovirus intervariant GII.4 recombinant in Victoria, Australia, June 2016: the next epidemic variant? Reflections and a note of caution

**DOI:** 10.2807/1560-7917.ES.2016.21.41.30372

**Published:** 2016-10-13

**Authors:** Jannik Fonager, Lasse Dam Rasmussen, Thea Kølsen Fischer

**Affiliations:** 1 Virology Surveillance and Research Section, Department of Microbiological Diagnostics and Virology, Statens Serum Institut, Copenhagen, Denmark

**Keywords:** viral infections, norovirus

**To the editor:** We wish to offer some cautionary remarks concerning the report by Bruggink et al. [1]. From an initial reading of the article, one could get the impression that the GII.P4_New_Orleans_2009_GII.4_Sydney_2012 recombinant form has only been possibly detected once before this study [2] and has – due to indicated novelty – a yet unknown pandemic potential. However, the GII.P4_New_Orleans_2009_GII.4_Sydney_2012 recombinant form has been reported earlier, both by us [3] in 2013 as well as by others [4,5]. The ORF1-ORF2 intergenic sequence (KX064756.1) submitted by the authors is almost identical (99.3%; 748 of 753 bp) to one of the sequences we submitted to the National Center for Biotechnology Information/GenBank in 2013 (KF199164.1), yet the authors only show separate phylogenies of the ORF1 fragment and capsid genes in their manuscript, masking the homology with previously published intergenic sequences.

We further consider it misleading that the authors do not mention that this recombinant form has been known to be in circulation since late 2012 and also that no phylogeny was presented based on alignments between their own ORF1/ORF2 spanning sequence (KX064756.1) and similar sequences from earlier studies. This gives the impression that no ORF1/ORF2 spanning sequences from the GII.P4_New_Orleans_2009_GII.4_Sydney_2012 are available in public databases, which indeed they are.

Also, since this recombinant contains the GII.4_Sydney_2012 capsid region (which is the most likely target for any acquired herd immunity), we find it unclear how recombination with a (internal) *pol* gene could be beneficial for the virus to escape the increasingly acquired herd immunity against the GII.4_Sydney_2012 capsid region.

Finally, we find that when the authors propose that the Sydney 2012 has a potential to become a new pandemic norovirus strain, it is highly important to also mention that it has been identified earlier and not give the impression that this is the first report about this recombinant strain.
